# An adaptable live-cell imaging protocol to analyze organelle morphology in *Saccharomyces cerevisiae*

**DOI:** 10.1016/j.xpro.2022.101124

**Published:** 2022-02-14

**Authors:** Pallavi Deolal, Krishnaveni Mishra

**Affiliations:** 1 Department of Biochemistry, School of Life Sciences, University of Hyderabad, Hyderabad 500 046, India

**Keywords:** Cell Biology, Genetics, Microbiology, Microscopy, Model Organisms

## Abstract

The protocol describes semiautomated live cell imaging in budding yeast. A key feature of the protocol is immobilizing cells in a culture dish, which allows for longer imaging times, changing culture media, or drug treatments. We describe steps for image acquisition and deconvolution, followed by manual analysis of quantifiable parameters to represent morphological changes in nuclear shape. We compare wild type with *ssf1Δ*, which is known to alter nuclear morphology. The protocol can be adapted to other organelles and processes.

For complete details on the use and execution of this profile, please refer to Male et al., 2020, Deolal et al. (2021).

## Highlights

• Proteins fused with fluorescent epitope tags can be used for monitoring organelle morphology
• Quantifiable parameters can be derived using FIJI/ImageJ
• Steps to image yeast nuclei and derive circularity index to compare morphology are described.
• The protocol can be adapted to derive measurements for other organelles

## Graphical abstract

**Figure:**
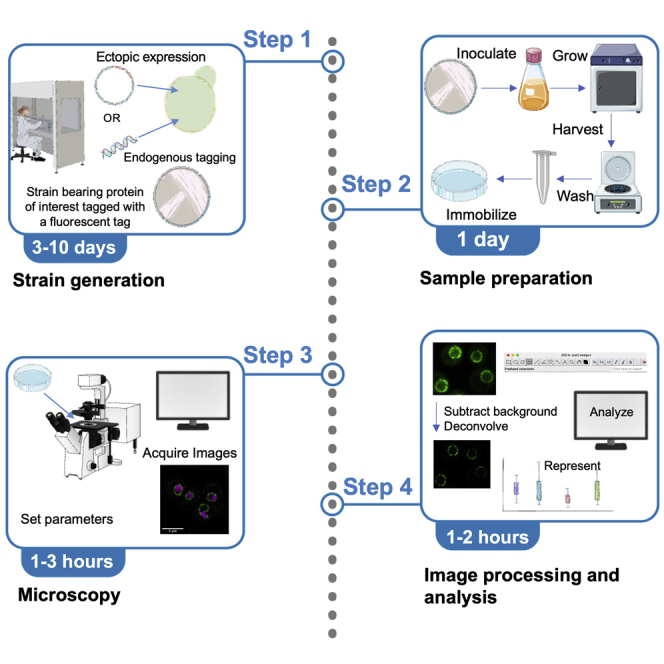

## Before you begin

The following is an easily adaptable imaging protocol for monitoring various structures and processes in budding yeast.

### Choosing a suitable fluorophore

**Figure 1:**
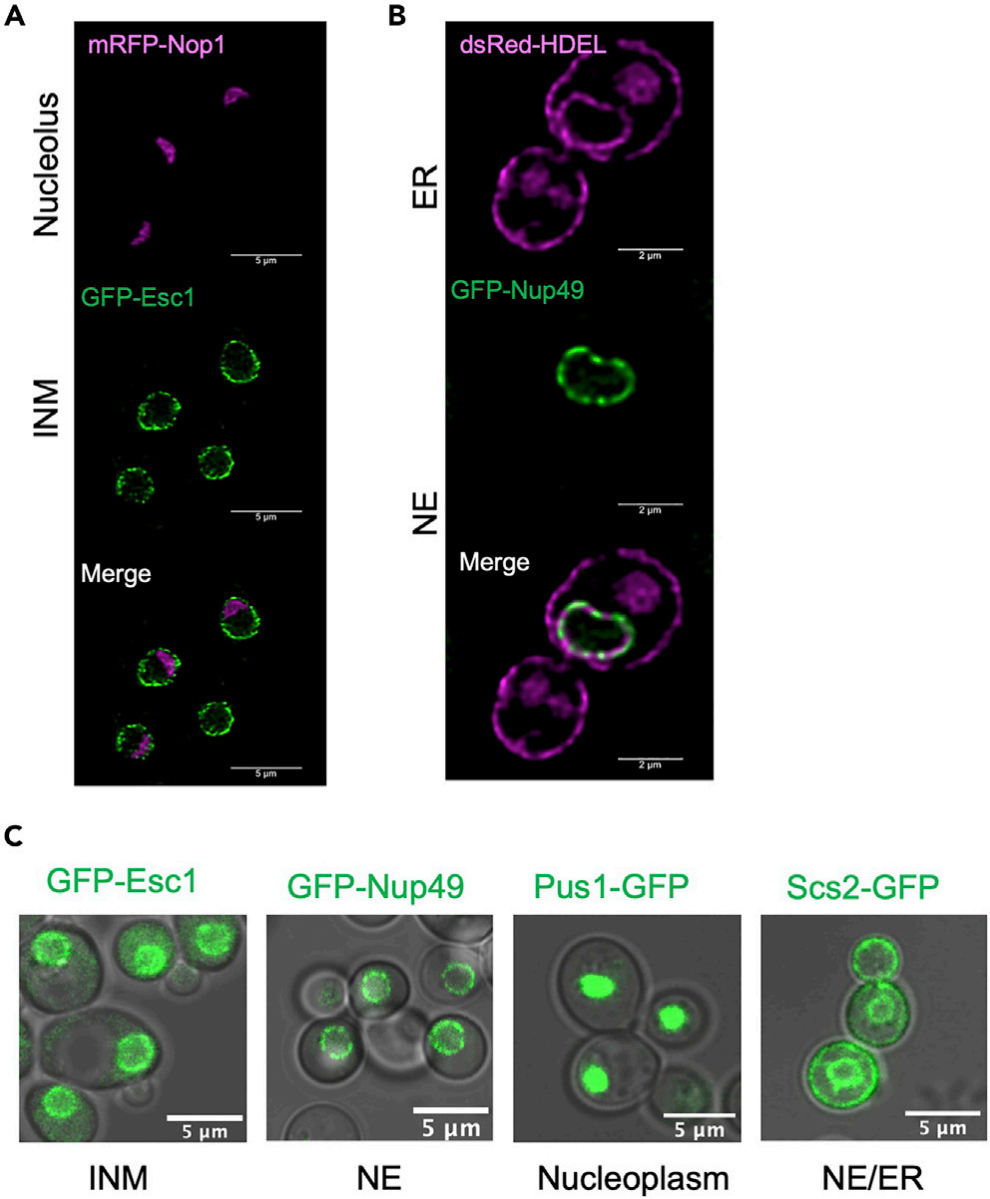
Representative images of nuclear components in budding yeast. The figure shows various nuclear sub-compartments in yeast imaged using ectopically expressed fluorescent markers in wild type cells (A) Nucleolus and nuclear membrane are visualized by mRFP-Nop1 and GFP-Esc1 respectively. Scale-5 μm. (B) dsRed-HDEL is used to mark ER and GFP-Nup49 marks the nuclear pore complexes distributed along the nuclear envelope. Scale-2 μm (C) The image panel shows localization of various proteins tagged with GFP that can be used to assess nuclear morphology. The nuclear region visualized using each construct is mentioned beneath the image. Scale-5 μm. Scale bar for each image is shown and micrographs in A and B are deconvolved using Huygens Professional.

Organelle morphology in live cells can be visualized by tagging a known protein with a suitable fluorophore Figure 1. Nucleolus in budding yeast appears as a crescent shaped structure at the nuclear periphery and occupies about one third of the nuclear volume (Figure 1A, magenta). The nuclear envelope (NE), a sub-domain of endoplasmic reticulum (ER) encircles the round nucleus (Figure 1A, 1B green). In budding yeast, the ER extends from the NE to the cell periphery and the two ring like structures are referred to as the perinuclear (pER) and the cortical ER (cER) (Figure 1B, magenta). Since each organelle has a defined morphology, geometric parameters can be derived from the images to compare the organelle morphology between samples. Commonly used parameters for defining morphology of the organelles are mentioned in Table 1.

***Note:*** Nuclei can be visualized by using various fluorescent markers (Tables 1 and 2). Here, we describe the method of obtaining images for budding yeast nuclei marked with an inner nuclear membrane (INM) targeted protein, Esc1, tagged with GFP.

**Table 1:** Proteins and parameters commonly used to assess organelle morphology in budding yeast

Organelle	Marker protein	Quantifiable parameter	ImageJ functions
**Nucleus**			

Chromatin	Htb1, Hht1	Roundness, Solidity, Volume	3D Objects Counter^a^, NucleusJ^a^
Nucleoplasm	Pus1		
Inner nuclear membrane	Esc1, Heh1, Heh2	Circularity index, Aggregation index or coefficient of variation	Analyze Particles, Plot profile
Nuclear pore complex	Nup49, Nsp1		
**ER**	Scs2, Kar2, Hmg1	Total surface area, Aggregation index or coefficient of variation	Analyze Particles, Plot profile
**Mitochondria**	Cox4, Tom70	Number, Length, Volume	3D Objects Counter^a^, MitoMap^a^
**Vacuole**	Vph1, Pho8	Roundness, Size	Analyze Particles, 3D Objects Counter^a^
**Peroxisomes**	Pex4, Pex1	Number, Average Size	Analyze Particles, Find Maxima
**Endosomes**	Sec7, Snf7		
**Lipid droplets**	Erg6, Tgl3		
**Plasma membrane**	Hxt1, Pma1	Total surface area, Intensity plot	Analyze Particles, Plot profile

a Plugins

**CRITICAL:** It is important to make sure that tagging does not compromise the normal function of the protein of interest. Functional assays can be performed to ensure the complementation. It is also best to ensure that introduction of an additional copy of the gene does not affect the function in case of plasmid-based approach. For instance, tagging Esc1 with GFP at the N terminus did not affect the role of Esc1 in establishment of telomeric silencing (Male et al., 2020).

**Table 2:** Common constructs with fluorescent tags and organelle targeting sequence

Organelle	Targeting sequence	Construct	References
Nucleus	SV40 large-T NLS	NLS-GFP	(Stade et al., 1997)
ER	Kar2	DsRed-HDEL	(Bevis et al., 2002)
Mitochondria	Subunit 9 of the F0-ATPase	Su9Mito-GFP	(Rapaport et al., 1998)
	Cox4	pHS12-mCherry	(Addgene Plasmid # 25444)
Peroxisomes	PTS1-SKL	pGFP-SKL	(Schäfer et al., 2004)

### Preparing the yeast strains by transformation of plasmid

**Timing: 3–10 days**

1. Choose the markers for visualizing the organelle of interest and prepare the strains. For using plasmid-based markers, transform the wild type (and other desired strains) with the plasmid(s). In this protocol, we have used a plasmid encoding GFP-Esc1 to image the yeast nucleus (Figure 1). Several commonly used fluorescent tags with localization signals are available and offer a simple alternative to visualize the organelle of interest. Some of these constructs are referenced in Table 2.

***Note:*** Ectopic expression of markers is a convenient approach for large screens. However, markers for assessing organelle morphology can also be expressed from an endogenous locus. Desired fluorescent tags can be integrated at the genomic loci of the protein of interest in multiple ways. Integration of the tag at the correct genomic site should be examined either by multiple screening PCRs or sequencing. Homologous recombination based strategies are widely used to tag genes either at N- or C-terminal (Longtine et al., 1998; Janke et al., 2004; Wang et al., 2017). Alternately, CRISPR/Cas9-based toolkits can also be used for tagging proteins or modifying existing libraries (Roggenkamp et al., 2017). While some of the endogenous tagging techniques allow marker-less genomic modification, the strains generated by homologous recombination at endogenous loci can also be used further without selection in Yeast extract-Peptone-Dextrose (YPD) medium or synthetic dropout media.

2. Restreak multiple transformed colonies. It is best to image cells from at least 3 independent colonies to assess the phenotype. The transformed strains should be grown in appropriate selection media based on the plasmid.

### Prepare the glass bottom dish for imaging

**Timing: 10–20 min**

The yeast cells are imaged in a 35 mm glass bottom dish (cover glass-12 mm diameter). The glass dishes allow closed-system imaging that prevents loss of media due to evaporation over longer durations of imaging (8–10 h). The dishes can also be placed in the incubator intermittently when images are required at a specific time duration. The cells can be given specific treatments such as change of media, addition of a drug or heat shock by conveniently taking the dish to a laminar air flow.

3. Prepare a 1% ConA solution by dissolving ConA powder in sterile, double distilled water. This stock can be stored in 4°C and diluted as per requirement.
4. Before imaging, the cells need to be immobilized by coating the base of the dish with 0.1% concanavalin (ConA).
  a. Spread 5–10 μL of 0.1% ConA solution over the cover-glass base with a pipette tip at room temperature (24°C) as shown in the video.
  b. Air dry the dishes for 5–10 min.

**CRITICAL:** Some protocols suggest storing ConA-coated slides and dishes. However, we find that freshly coated dishes work best. It does not take more than 5–10 min for air drying if spread well. Also, longer storage of cover glass or dishes results in dust / dirt settling on the surface, which can affect image quality (See troubleshooting Problem 1).

***Alternatives:*** Instead of ConA, a 0.1% poly-L-lys (P8920-Sigma) can also be used to coat the cover glass for immobilization of cells. The glass-bottomed dishes are also available as multiwell dishes (IBIDI-80427). The cells can be adhered to a cover glass, as mentioned above, and mounted over the imaging platform. Open confocal imaging chambers (RC-30 Confocal imaging chambers-Warner instruments, A7816 Attofluor^TM^ cell chamber- Invitrogen^TM^) can be used instead of glass dishes.

***Note:*** Reusing the culture dish: We have been regularly reusing the dishes (at least 4–5 times) to image cells, without compromising the quality of images. In order to reuse, wipe off the immersion oil from the base of the dish after first use. Remove the medium from the dish and soak the dish in a mild dish-wash detergent for 5–10 min followed by thorough wash in running water. Rinse twice with sterile distilled water. To ensure that the glass is clean, spray 70% ethanol and wipe the dish gently with lens cleaning tissue once. Let it dry. If longer storage is required, further sterilization can be done using UV light.

## Key resource table

**Table:** 

REAGENT or RESOURCE	SOURCE	IDENTIFIER
**Chemicals, peptides, and recombinant proteins**		

Yeast Nitrogen Base without amino acids	Difco	Cat no#291940
D-(+)-Glucose	Himedia HiMedia	Cat no#GRM077
Agar Agar, Type I	Himedia HiMedia	Cat no#GRM666
L- Arginine Sulfate	Sigma-Aldrich (Merck)	Cat no#A8094
L-Glutamic Acid	Sigma-Aldrich (Merck)	Cat no#G8415
L-Histidine	Sigma-Aldrich (Merck)	Cat no#H5659
L-Isoleucine	Sigma-Aldrich (Merck)	Cat no#I7403
L-Leucine	Sigma-Aldrich (Merck)	Cat no#L8912
L-Methionine	Sigma-Aldrich (Merck)	Cat no#M5308
L-Phenylalanine	Sigma-Aldrich (Merck)	Cat no#P5482
L-Tryptophan	Sigma-Aldrich (Merck)	Cat no#T0254
L-Tyrosine	Sigma-Aldrich (Merck)	Cat no#T8566
L-Valine	Sigma-Aldrich (Merck)	Cat no#V0500
Adenine Sulfate	Sigma-Aldrich (Merck)	Cat no#A2786
Uracil	Sigma-Aldrich (Merck)	Cat no#U1128
Concanavalin A	Sigma-Aldrich (Merck)	Cat no#C2010

**Experimental models: Organisms/strains**		

*S. cerevisiae* Wild type MATa his3Δ1 leu2Δ0 met15Δ0 ura3Δ0	Euroscarf	BY4741 (Baker Brachmann et al., 1998)
*S. cerevisiae* ssf1::KanMX MATa his3Δ1 leu2Δ0 met15Δ0 ura3Δ0	Euroscarf	n/a
*S. cerevisiae* yop1::KanMX MATa his3Δ1 leu2Δ0 met15Δ0 ura3Δ0	Euroscarf	n/a

**Recombinant DNA**		

GFP-Esc1-pDZ45	(Male et al., 2020)	n/a
Pus1-GFP-pUG23	(Male et al., 2020)	n/a
GFP-Nup49-pUN100	(Belgareh and Doye, 1997)	n/a
mRFP-Nop1-pRS316	(Ulbrich et al., 2009)	n/a
dsRed-HDEL-yIplac204	Karsten Weis (Institute of Biochemistry, ETH Zurich)	pKW1358
Scs2-GFP-pRS416	Chris. J Stephan (Laboratory for Molecular Cell Biology at University College London)	n/a
pHS12-mCherry	Addgene	Plasmid #25444

**Software and algorithms**		

LAS X (Version: 3.5.7)	Leica Microsystems CMS GmbH	n/a
FIJI (Version: 2.1.0/1.53c)	Open Source Software (Schindelin et al., 2012)	n/a
Huygens Professional (Version: 17.04)	Scientific Volume Imaging B.V.	n/a
Microsoft Excel (Version: 16.53)	Microsoft	n/a

**Other**		

50 mL tube	Corning	Ref# 430829
1.5 mL microfuge	Eppendorf	Ref# 500010
Petridishes	Falcon	Ref# 353147
Millex-GV Filter Unit 0.22 μm	Millipore, Merck	Ref# SLGV033RS
Incubator shaker, Innova 42	New Brunswick Scientific	n/a
Centrifuge 5420	Eppendorf	n/a
Nanodrop 2000C	ThermoScientific	n/a
Leica TCS SP8	Leica	n/a
Nunc glass base dish	Thermo Scientific	Cat no#150682
Stage Top incubator	OKO Lab	n/a
Type F Immersion Oil	Leica	CAS no#195371-10-9
Lens cleaning tissue (M97)	Olympus Optical Co. Ltd.	Ref# AX6476

## Materials and equipment

### Preparation of powder omission media mix

To make SC dropout mix, mix all the ingredients mentioned below using a blender. Depending on the auxotrophic selection, exclude the amino acid powder which is not to be included.

**Table:** SC Dropout mix

Reagent	Final concentration	Amount
Yeast Nitrogen Base without amino acids	1×	50 gm
L- Arginine Sulfate	20 mg/L	150 mg
L-Glutamic Acid	100 mg/L	3.75 gm
L-Histidine HCl	20 mg/L	150 mg
L-Isoleucine	30 mg/L	225 mg
L-Leucine HCl	100 mg/L	450 mg
L-Methionine	20 mg/L	150 mg
L-Phenylalanine	50 mg/L	435 mg
L-Tryptophan	20 mg/L	150 mg
L-Tyrosine	30 mg/L	225 mg
L-Valine	150 mg/L	1.12 gm
Adenine Sulfate	20 mg/L	150 mg
Uracil	20 mg/L	150 mg

**Storage:** Powder can be stored at room temperature (22°C–26°C) for up to 1 year.

***Alternatives:*** Mortar and pestle can be used instead of a blender to mix the powder ingredients. Ready-made dropout mix can be purchased commercially.

**Table:** 100 mL growth medium

Reagent	Final concentration	Amount
SC (dropout/complete)	0.75%	0.75 gm
Dextrose	2%	2 gm
Agar (for plates only)	2%	2 gm

**Sterilize the media by autoclaving at 121**°**C for 20 min.** For plates, set pH to 5.8–6.2 using drops of 10N NaOH solution, otherwise the agar will not solidify properly.

**Storage:** Media can be stored at room temperature (22°C–26°C) for up to 6 months.

***Note:*** Synthetic complete (SC) media is suitable for imaging due to low autofluorescence in comparison to YPD, we recommend growing the primary culture in SC media to avoid any undesirable metabolic changes occurring in the cell due to differences in growth medium and imaging medium.

## Step-by-step method details

### Preparation for growing and imaging cells

**Timing: 1–2 days**

**Figure 2:**
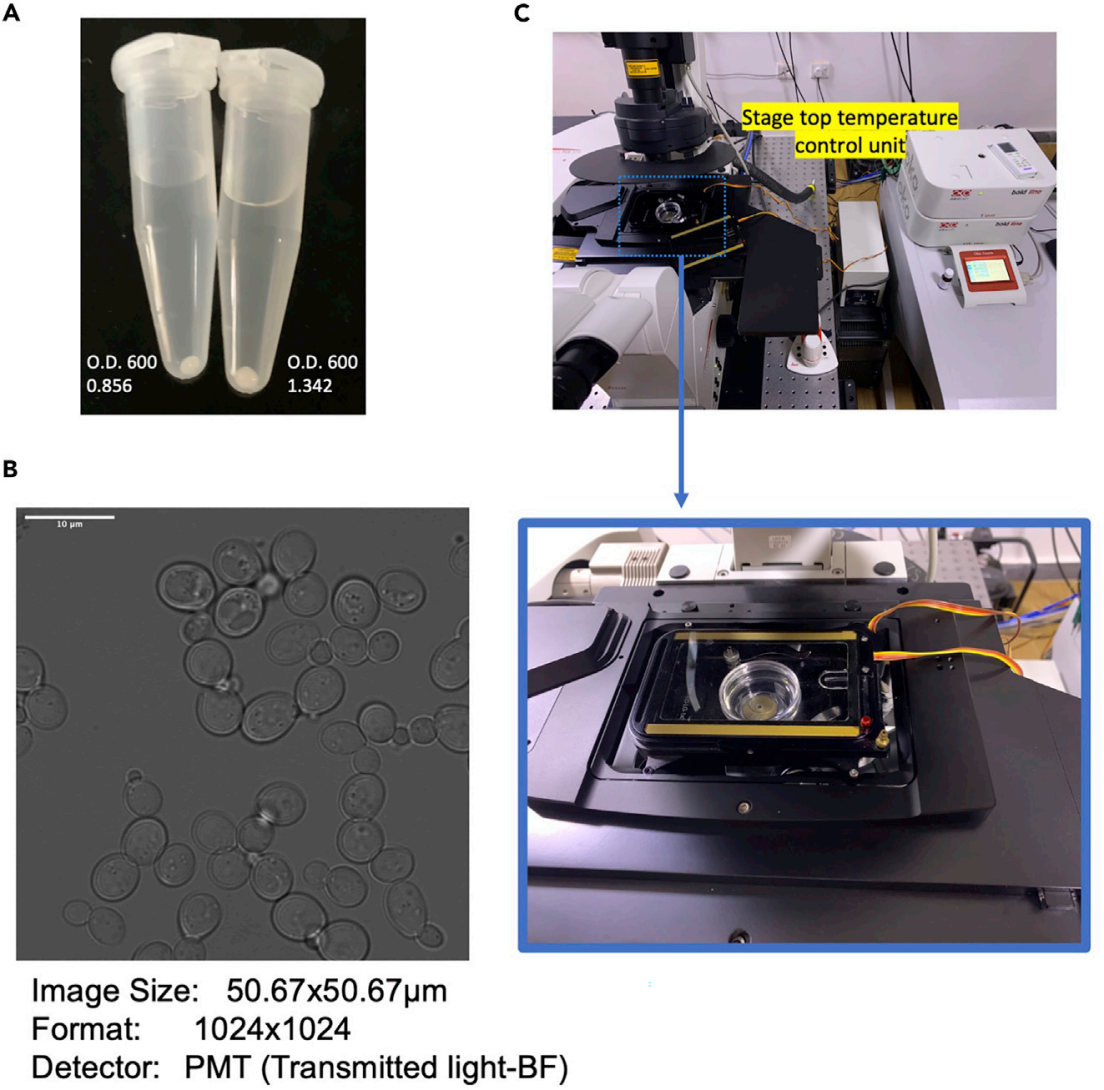
Preparing cells for imaging (A) The picture shows pellet size obtained for cells harvested at the indicated OD600. (B) The field represents an ideal distribution of cells for acquiring images. Scale-10 μm (C) The image shows a live-cell imaging setup with a 35 mm dish with glass bottom mounted on a temperature-controlled stage.
  b. Wash the pellet with 1 mL of fresh media prewarmed to the growth temperature (usually 30°C). This allows removal of any debris or particles that can interfere with the field of view while imaging.
  c. Resuspend the pellet in 20–50 μL of media.

1. Grow the liquid cultures
  a. Inoculate a single colony of yeast in appropriate synthetic minimal/ complete media and grow overnight (12–14 h) at 30°C with shaking at 220 rpm.
  b. Sub-culture into fresh medium to a starting OD 600 of 0.15–0.2 the next day and grow for 3–4 h at 30°C with shaking at 220 rpm.
2. Prepare the cell suspension
  a. Harvest cells from mid-log phase (OD 600- 0.6 to 1.0) by spinning 1 mL of culture at 3000g for 1 min. If imaging from other stages of growth, use appropriate volume of the starting culture. For reference to the size of pellet, see Figure 2A.

3. Immobilize the cells (Methods video S1)
  a. Take the glass bottom dish coated with 0.1% ConA.
  b. Lay 10 μL cell suspension over the ConA coated dish and spread gently with P200 pipette tip.
    ***Note:*** Immobilization of cells with 0.1% ConA in the glass dish containing growth media does not affect the cell division time. The wild-type cells complete division in 80–100 min, similar to the time taken when grown in a liquid culture placed in an incubator.
  c. Let the cells adhere to the glass by leaving it undisturbed for 3–5 min.
  d. Add 500 μL of media and mix up and down gently using a pipette to remove unadhered cells. Remove excess liquid and repeat the process two times.
    **CRITICAL:** Do not pipette directly over the cells to avoid loss of adhered cells.
  e. Add 500 μL -1 mL of fresh media and begin imaging.
    ***Note:*** The above steps give a cell density of approximately 35–50 cells in a field of 50 μm × 50 μm (Figure 2B). The cells remain healthy for up to 12 h in media containing 2% glucose. In order to increase cell density, a higher volume of cell suspension can be laid over the ConA coated base. To reduce the cell density, either dilute the cell suspension or increase the washing step mentioned in step 6d. For dishes with a glass base of 25 mm diameter, use 20 μL ConA (0.1%) to coat the base. Similarly, a higher volume of cell suspension can be laid on the coated base (30–50 μL).
  Table S1. Excel sheet with values of shape descriptors exported from FIJI, related to step 7e

### Image acquisition

**Timing: 30 min**–**3 h**

Here we describe the steps involved in acquisition of images. Some of the terms and features mentioned are specific to acquiring images on Leica TCS (SP8) using LAS X. However, equivalent options also exist in other commonly available microscope devices and their respective software. They have been mentioned where necessary. We use a HC PL APO CS2 63×/1.40 OIL objective on our Leica TCS SP8. The temperature is controlled using a stage top incubator (Figure 2C). For standard wild type yeast, the temperature is set to 30°C.

**Figure 4:**
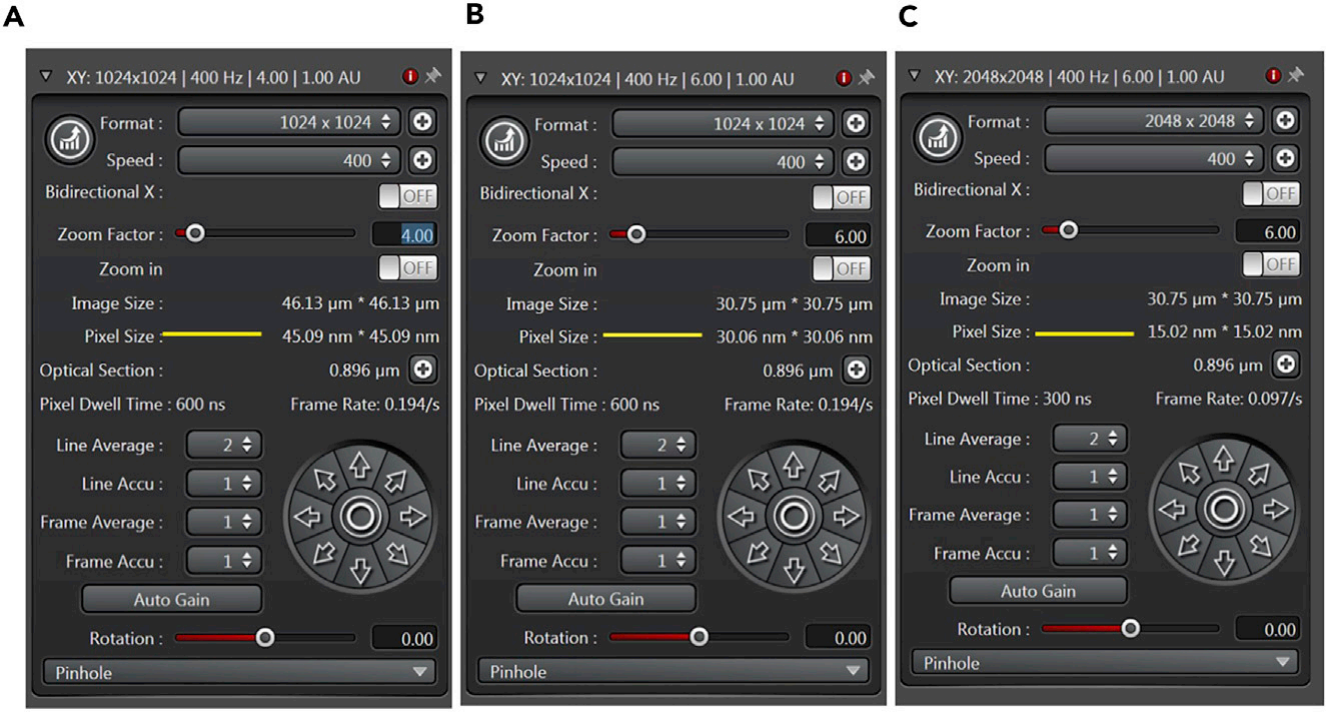
Setting image acquisition parameters (A–C) The figure shows a comparison between three different settings for image acquisition. Pixel size can be reduced either by imaging at a higher format or increased zoom. Settings in panel A and B have the same acquisition format and speed, but the pixel size in B is smaller due to increased zoom. For the same field size in B and C, the pixel size in C is lower due to a higher imaging format (2028 × 2048 in C versus 1024 × 1024 in B).
  b. To semi-automate the process of image acquisition, positions of multiple fields can be defined by registering the XY coordinates in the acquisition mode. The ‘Mark and Find’ function of LAS X can be used as shown in Figure 5A. Multiple positions can be easily defined in other softwares also (locate ‘Positions’ on Multi-dimensional acquisition mode in Zen for Zeiss and ‘Process manager’ in CellSens for Olympus).
    **CRITICAL:** To avoid drift, activate the autofocus function (Figure 5B). This ensures that each position is detected and imaged accurately in the selected z-axis. Also, for automated imaging it is best to store positions that are near-by. Moving too far in the field of view while navigating costs time and can result in xy-focal drift overtime.

4. Set the imaging parameters
  a. Place the dish on the stage after adding immersion oil to the objective and focus a field of cells.
    **CRITICAL:** If several rounds of cell division are to be recorded during time lapse imaging, start with a field with low cell density (3–5 cells/50 μm × 50 μm field).
  b. Visualize the signal for the fluorophore of interest. During visualization and scanning for a proper field of view, a low imaging format (512 × 512 or 1024 × 1024) can be used with a high scan speed (400 Hz or 600 Hz). Set the laser intensity and detector gain, and observe the signal.
    ***Note:*** Start scanning with low laser power (0.5%–2%) and increase further based on the signal. The laser output can vary depending on laser power and usage lifetime. Ensure that the signal is not saturated by following the image histogram to check the dynamic range. Switch to ‘Quick lookup table’ mode on Leica to highlight saturated regions. Equivalent ‘Range indicator’ is also available in Zen while using the Zeiss microscope.
    **CRITICAL:** Ensure that the cells have adhered properly and there are no cells floating in the field of view. If the field of view is crowded with cells or cells are layered, repeat step 6d until optimum density is obtained (Compare 2B and 3A). Alternately, the cell suspension can also be diluted before placing cells for immobilization. Ensure that there is no bacterial contamination and the background should also be clear (Figures 3B and 3C). If a lot of nonspecific signals appear, refer to troubleshooting steps Problem 1 and 2.
    **Figure 3:**
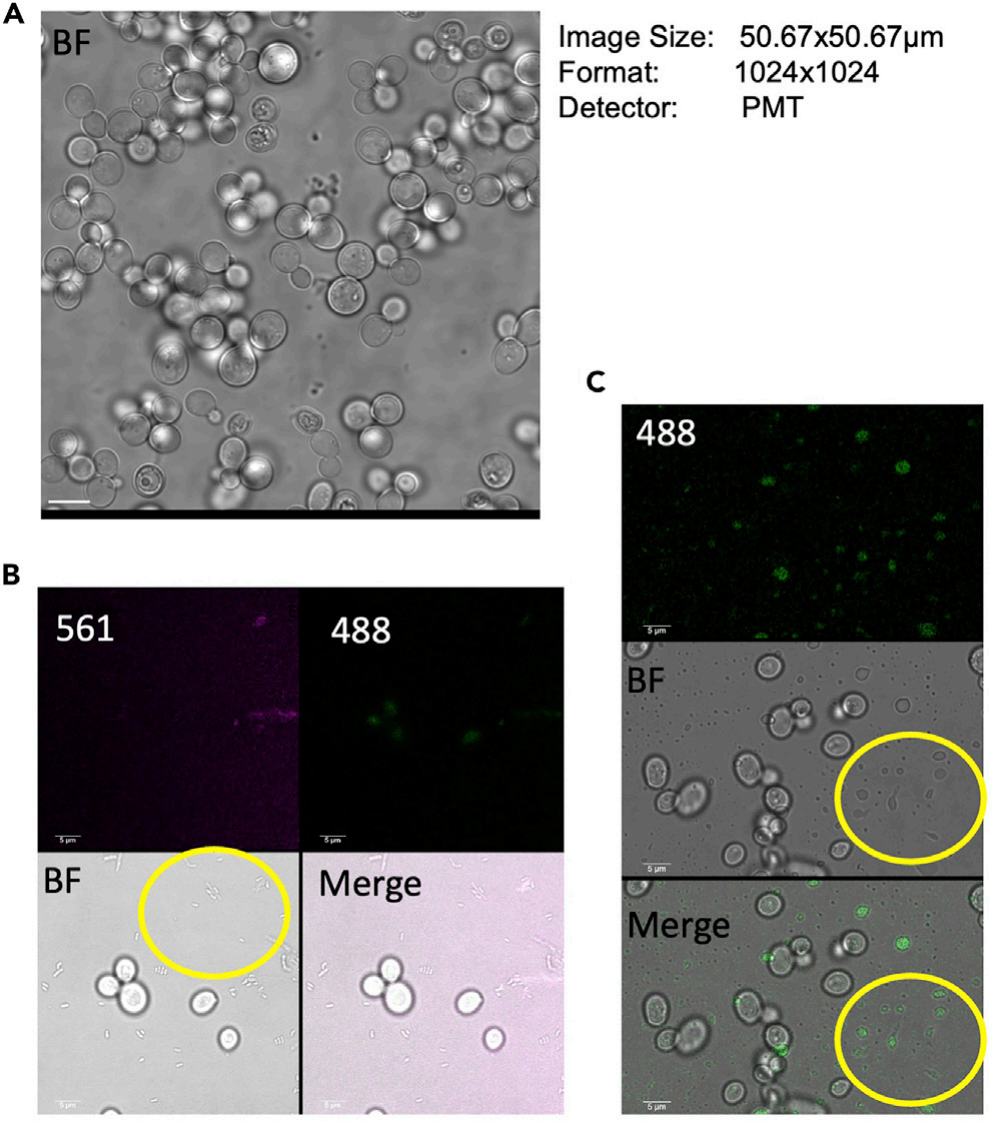
Fields to avoid during imaging (A) This micrograph shows a field which is populated with too many cells. Such regions should be avoided at the start of a time course of live cell imaging. Scale-10 μm (B) The micrograph shows bacteria contaminating the culture. The plane shows bacterial culture in focus. Scale-5 μm (C) This image shows non-specific fluorescent signals due to contaminating particles in the imaging medium. Scale-5 μm

5. Acquire images
  a. Choose the correct format and image size. Figure 4 shows how different image formats can affect the pixel size. Line averaging of 2–3 can be used with frame accumulation to improve the quality of image and signal to noise ratio (SNR). We advise using the ‘optimal’ z-step size suggested by the software based on the image format and size. This is an in-built feature on most of the commercially available microscope software. Basic settings used for image acquisition of GFP and RFP tagged proteins in *S. cerevisiae* at 400 Hz scan speed are mentioned hereunder:

**Table:** 

Function	GFP	RFP
Laser	488 nm	543 nm/561 nm
Laser Intensity	0.5%–1.0%	1.0%–1.5%
Detector	HyD^TM^	HyD^TM^
Detector Gain	310–350	350–400

    ***Note:*** Image acquisition time varies based on the requirement. Faster processes such as protein and organelle dynamics can be recorded with shorter time intervals (5–10 s), while monitoring long lived proteins and slower processes over the course of cell division will take longer time (3–5 min). Pixel size of the image should be at least 2.3 times smaller than the structure being resolved (Nyquist Sampling). This will vary based on the image resolution and size, and objective in use. For instance, pixel size of 40–50 nm is sufficient for gross morphological observation of yeast mitochondrial or nuclear morphology. However, for structures such as autophagosomes or ER-mitochondria contact sites, images with 10–20 nm pixel size should be acquired. Most of the microscope operating systems have an in-built calculator to determine the optimum sampling. Ideal sampling can also be calculated using online tools such as https://svi.nl/NyquistCalculator. Even in a xyz scan, one must select the step size determined optimally. While monitoring processes, the imaging rate should also be 2.3 times faster than the actual process. Therefore, the time interval between images should also be determined carefully. When doing longer time course imaging, under sampling is preferable to get a brighter signal and prevent photobleaching. Oversampling implies increased acquisition time, reduced signal intensity and higher photobleaching.

**Figure 5:**
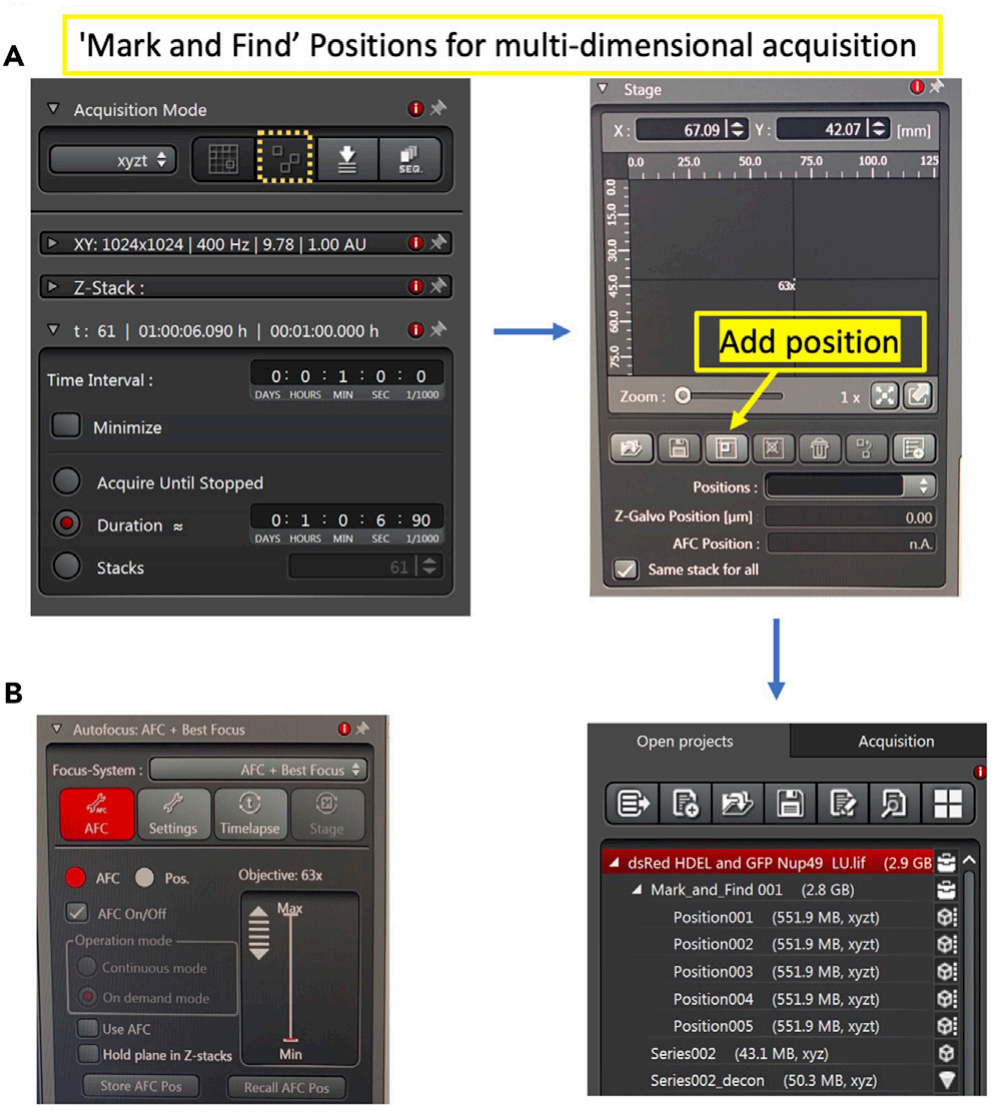
Marking fields for imaging (A) The yellow box with a dotted line highlights the ‘Set Mark and Find’ option in the LAS X Acquire tab. Activating this opens a dialog box where stage coordinates can be seen. Multiple positions can be saved while scanning the field for regions to be imaged. Once the acquisition is started, the images corresponding to each position are saved under Mark and Field in the active project. (B) In order to prevent drift during longer imaging, the autofocus function can be used. A mid-focal plane can be stored as an AFC position at the start of acquisition.
  c. Once the regions are marked and stack thickness and step size are set, begin the acquisition. A project tree is generated that stores images for each position marked.
  d. The images can be exported directly to Huygens Professional for deconvolution or processed later after acquiring all the samples.
    **CRITICAL:** Imaging 3–4 fields, yields sufficient cells (∼120 cells) for manual phenotypic assessment per replicate. One must image at least three biological replicates for each sample.

### Deconvolution and analysis of morphology parameters

Here we describe the steps involved in deconvolving images using the deconvolution wizard of Huygens Professional version 17.04 (Scientific Volume Imaging, The Netherlands, http://svi.nl). Huygens computes a theoretical PSF (point spread function) to restore the images based on the imaging parameters that include the kind of microscope and acquisition parameters (viz image size, pixel size, z-step size). Alternatively, deconvolution can also be performed using in-built deconvolution tools of respective microscope software or plugins for ImageJ/FIJI (Deconvolution, DeconvolutionLab2).

**Figure 6:**
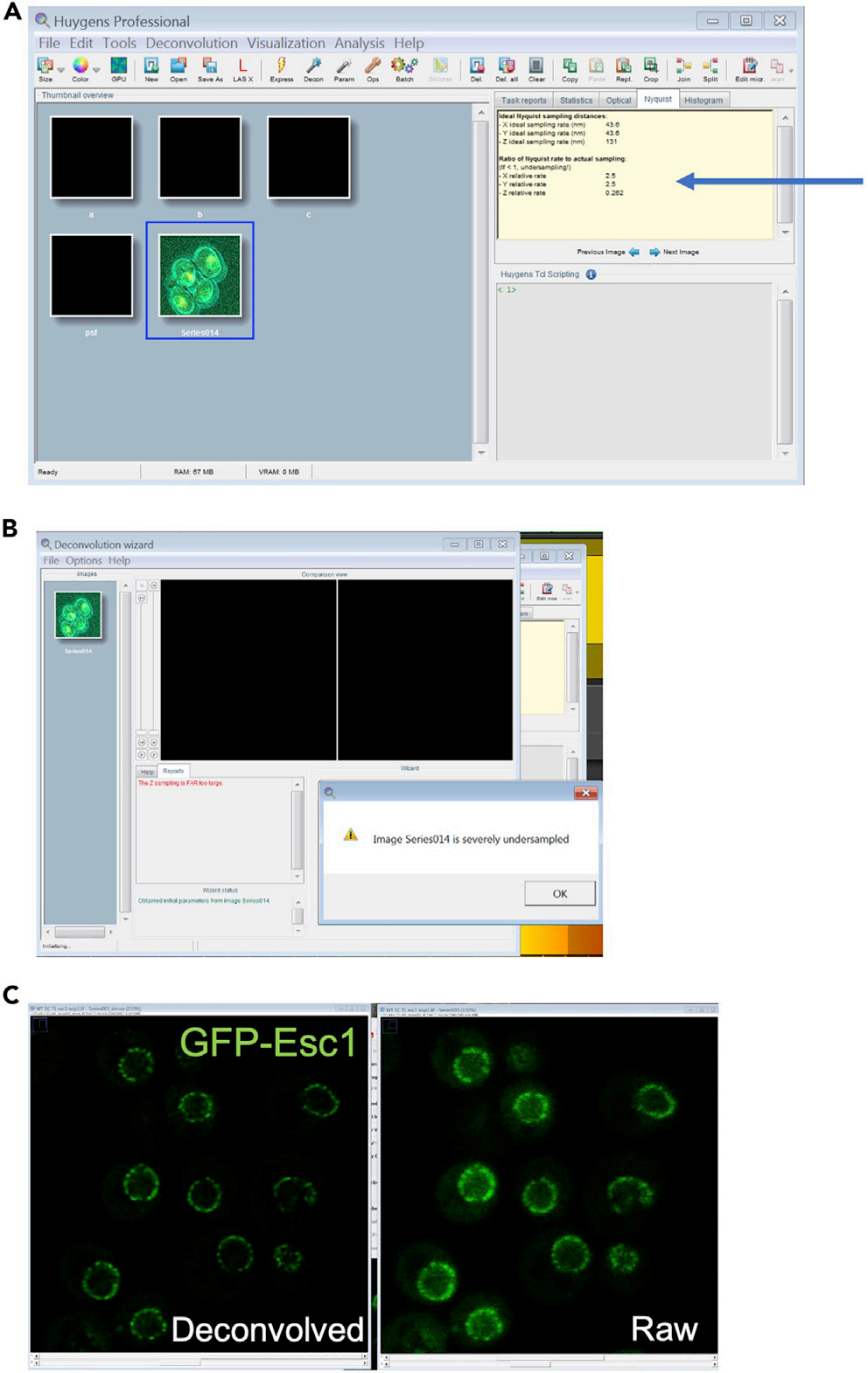
Deconvolution using Huygens Professional (A) Images acquired using LAS X can be directly exported to Huygens Professional or opened using import file function. The metadata of the image is read by the deconvolution software. Features of the image in selection can be read in the tab. The arrow in A shows the Nyquist sampling parameters. (B) If the imaging parameters are incorrect, the deconvolution wizard shows a message indicating either oversampling or under sampling. (C) The two screenshots show nuclear envelope of wild type cells marked by GFP-Esc1 after and before deconvolution.
  c. Before starting deconvolution, check the image quality. The Nyquist parameters can be viewed for the image under selection (arrow, Figure 6A).
  d. Right click on the selected image and select deconvolution wizard. If the image acquisition parameters are not optimum, a dialog box with the issue will appear (Figure 6B). Use another image obtained at a higher format and better resolution.
  e. The subsequent steps involve an automatic estimation of the background done using the in/near object estimation mode. GMLE (Good’s roughness Maximum Likelihood Estimation) deconvolution algorithm is used and images are deconvolved on the basis of PSF in an iterative fashion. The signal to noise ratio (SNR) can be set between 10–14 depending on the intensity of fluorophore.

6. Deconvolve the images.
  a. Export the images to Huygens professionals.
  b. The thumbnail of exported images can be viewed in the window (Figure 6A).

The effect of deconvolution on NE staining using the GMLE algorithm, with SNR:12 and 10 iterations is shown in Figure 6C.

**Figure 7:**
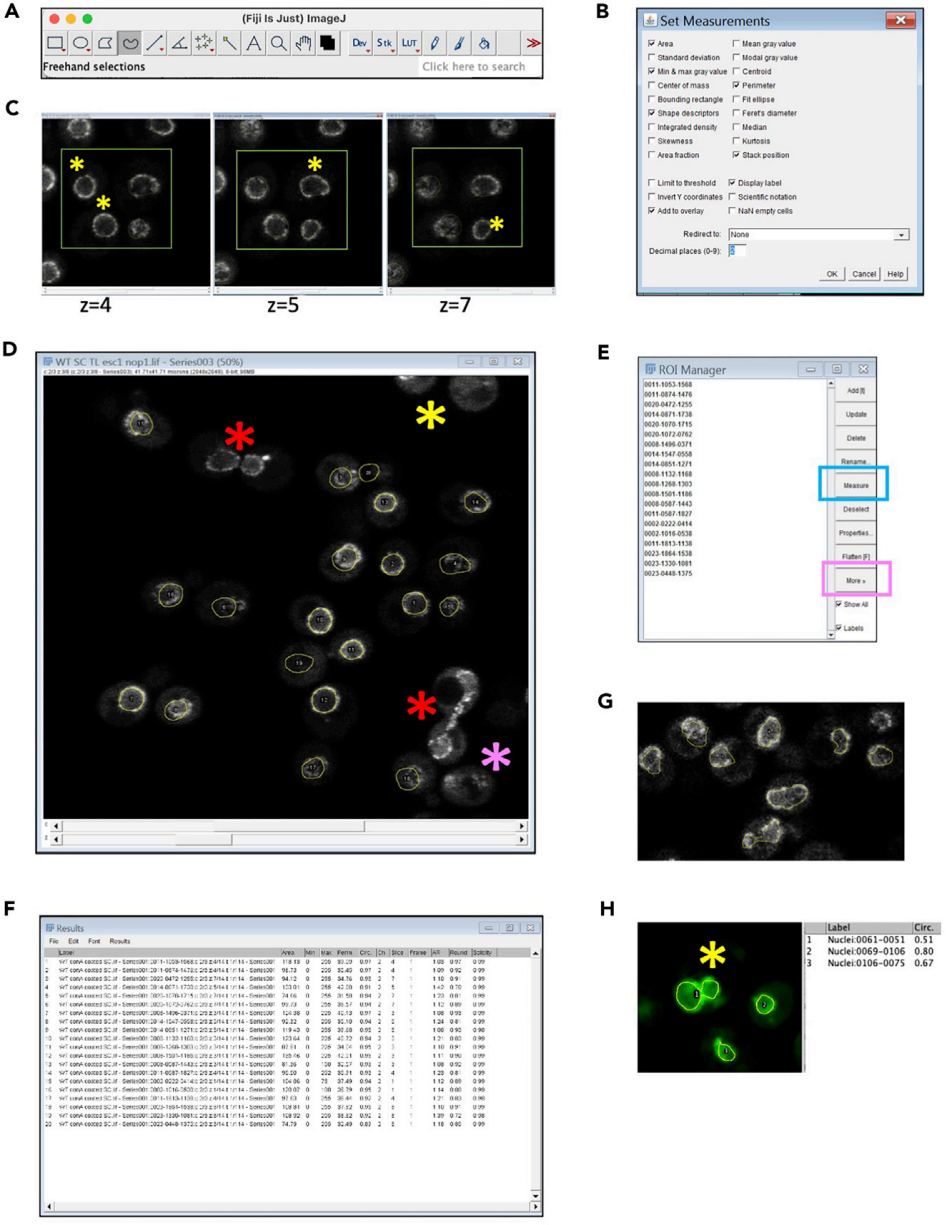
Image analysis using FIJI (A) FIJI toolbar with the freehand tool in selection is shown here. (B) The Set Measurement window showing parameters that can be measured for a selection is shown. To access this window- Click on analyze menu from the menu bar→ select Set measurement from the drop-down menu. (C) In order to get measurements for each nucleus, the objects have to be marked manually. The freehand tool is used to draw over the nuclear outline in the focal plane. The plane with maximum diameter for each nucleus can vary. The star marks the nuclei for which maximum diameter can be seen in the mentioned z-stack. For the 4 nuclei within the region marked in green box, the yellow star indicates the nucleus for which outline is marked in the indicated z-plane. (D) The star mark indicates the nuclei not considered for phenotype assessment in this panel showing nuclei of wild type cells. (E and F) After all the objects have been marked, the ROI manager shows a list of those objects. Click on Measure (Blue rectangle) and this gives a results window as shown in (F). This indicates the values of all the parameters selected in B. The values can be exported for further analysis. (G and H) A screenshot of cells with abnormal nuclear morphology marked for measurement.
  b. To select the desired measurements, go to the analyze menu and select Set Measurement. Parameters of interest can be selected and used for further comparison (Figure 7B). Select ‘Shape descriptors’ option to get circularity index.
  c. Trace the nuclear outline, circumscribing the Esc1-GFP signal using the free hand selection tool (Figure 7C).
    **CRITICAL:** Adjust the pixel width of the line depending on the structure that is traced. To change the thickness of the selection, select the ‘Freehand selections’ tool, go to the Edit menu, click on Options and enter the appropriate value.
    ***Note:*** Not all cells in the field will have the nuclei in the same plane. Therefore, the z section should be selected carefully before drawing the ROI (Region of interest) over the NE. As shown in Figure 7C, the maximum nuclear perimeter lies in different z-planes for the indicated nuclei marked with star.
  d. After marking each nuclei, add the selection to the ROI manager (Press T or go to Edit in the menu bar → Selection→Add to Manager). Ensure that the ‘Label’ box is ticked in the ROI manager dialog box to avoid marking the same nuclei multiple times. (Figures 7D and 7E)
    **CRITICAL:** When marking the nuclei, we excluded dividing cells (red ∗ in Figure 7D), cells with entire nucleus not covered in the stack (Magenta ∗ in Figure 7D) or cells in the edges of field (Yellow ∗ in Figure 7D).
  e. After completing selection of cells, click on Measure and the Results tab appears as shown in Figure 7F. Save the table as either .csv or copy the data and import to Excel (Table S1: Tab1-Circularity index)

7. Quantify the morphological parameters using FIJI
  The nuclear morphology between strains can be compared by assessing the distribution of a nuclear envelope marker. The Circularity Index is one of the most widely quantified morphological features of the nucleus. It is calculated as: 4∗π∗area/perimeterˆ2. The steps below are a guide to measure the circularity of the nucleus. Other measurements such as perimeter, area and volume can also be extracted using this method by the Analyze tool of FIJI (Schindelin et al., 2009).
  a. Open the image file in FIJI (Figure 7A).
    **CRITICAL:** For comparison between two samples, images of the same format and size should be used.

**Figure 8:**
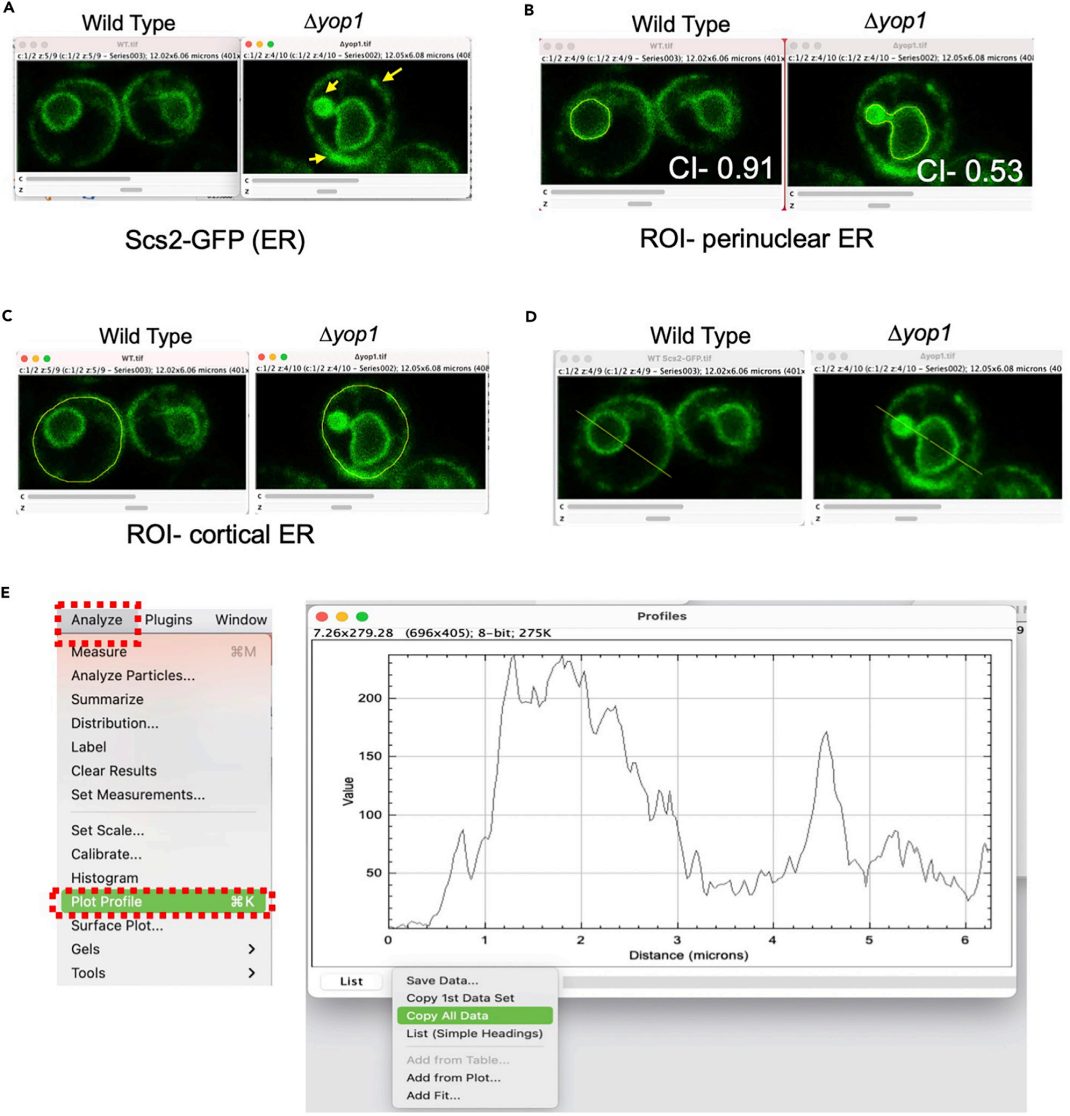
Analyzing ER morphology (A) The image shows ER morphology as marked by Scs2-GFP for wild type (left) and *yop1Δ* (right). The abnormalities observed in *yop1Δ* are marked by yellow arrows. (B) The screenshot shows selection (yellow outline) of the perinuclear ER for wild type (left) and *Δyop1Δ (right)* using the freehand tool. The circularity index (CI) varies between the two samples, indicative of the phenotype. (C) The screenshot shows selection (yellow outline) of the cortical ER for wild type (left) and *yop1Δ (right)* using the freehand tool. (D) The screenshot shows the line (yellow line) drawn across the wild type (left) and *yop1Δ (right)* cell drawn using the line tool. (E) The intensity profile for the ROI can be obtained by selecting the ‘Plot profile’ option from the ‘Analyze’ menu as shown in the screenshot. This gives a plot as shown on the right. Click on ‘Data’ and then copy all data to excel for analysis. The data can also be saved as a .csv file by clicking on ‘Save Data’ option (Blue arrow).

**Analysis using ER membrane marker:** Similar analysis can also be performed to quantify the differences in the distribution of ER localized proteins. An example of this is presented in Figure 8. Here we compare the differences in ER architecture between wild type (Figure 8A, left) and *Δyop1* (Figure 8A, right). Yop1 is an ER protein involved in tubule formation. Since the perinuclear ER (pER) outlines the NE, circularity of the pER can be measured to represent phenotypic variations between samples (Figure 8B). The distribution of the marker protein along peripheral or cortical ER (cER) can also be determined similarly by selecting a ROI using freehand tool (Figure 8C, Table S1: Tab2-Plot profile). To compare differences in distribution of protein between pER and cER a line profile of fluorescence intensity can be plotted by drawing a line across the cell using ‘Line Tool’ as shown in Figures 8D and 8E. Such line profiles for multiple ROIs can be extracted from the ROI manager by clicking on More, followed by Multi Plot (Figure 7E, magenta outline). The intensity based differences can be further quantified as aggregation index (Lord and Wente, 2020; Deolal et al., 2021) or a function of variation in intensity along the ROI (Schuck et al., 2009). Intensity profiles from at least 10 to 15 cells from 3 independent experiments should be compared to derive inferences on phenotype.

**Figure 9:**
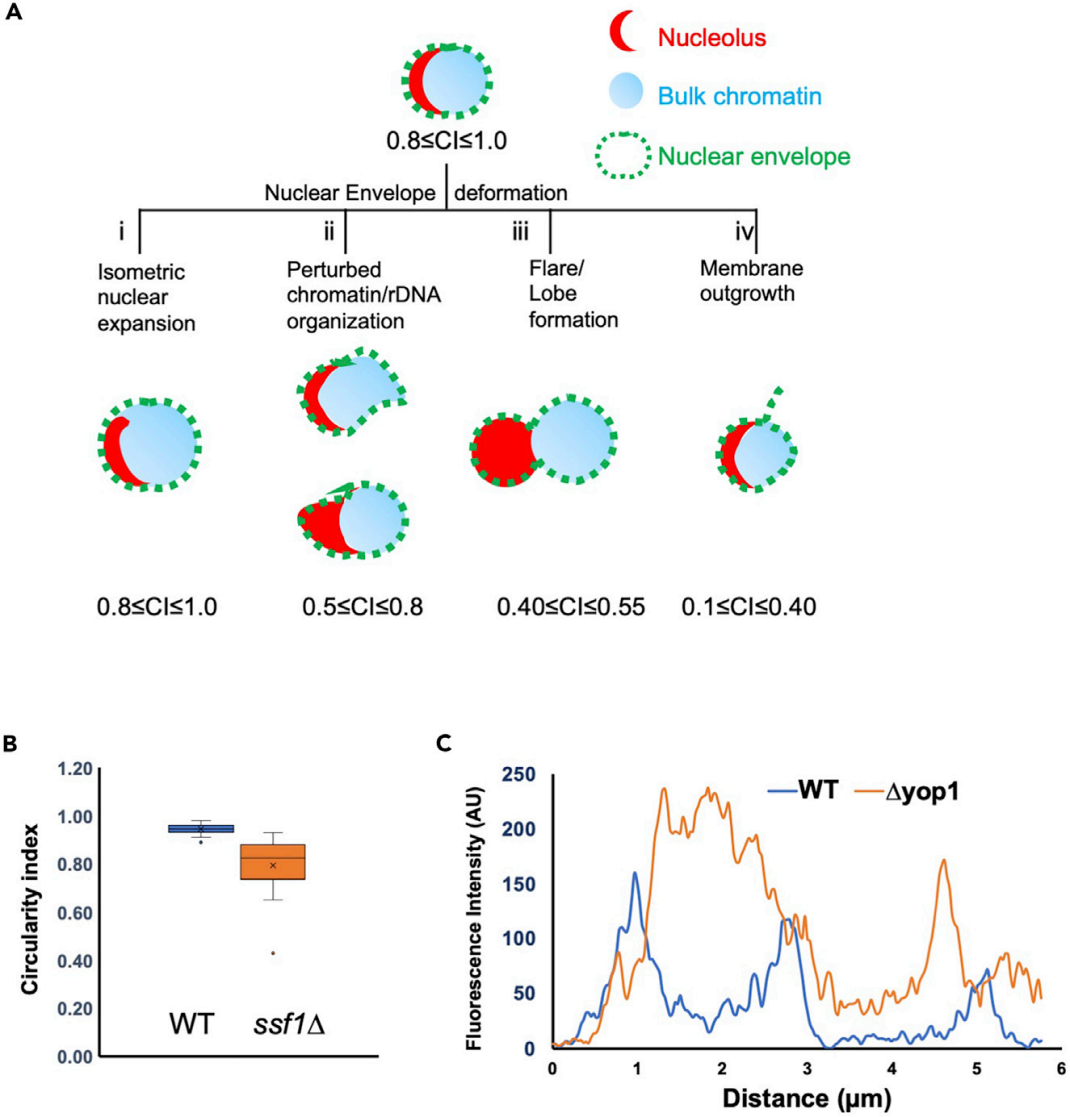
Analysis and quantification (A) The cartoon represents expected outcome when calculating circularity index of nuclei using a NE marker (green). The circularity index of nuclei reduces as the deformities increase. For most spindle shaped or bilobed nuclei, the circularity index value lies close to 0.5 if derived by tracing NE outline. (B) The box and whisker plot shows a comparison of the circularity index between wild type and *ssf1Δ* cells. Horizontal line at the center represents the median (Table S1: Tab1-Circularity index). The narrow range of distribution for wild type shows that most of the cells display a round nuclear morphology unlike the abnormal shapes seen in *ssf1Δ.* (C) The plot shows the intensity-based profile for WT and *yop1Δ* as marked in Figure 8D (Table S1:Tab3-Line profile). The localization density of Scs2-GFP spans a broader area representative of the perinuclear ER extension for *yop1Δ* as shown in Figure 8D, *right.*

***Alternatives:*** Nuclear shape can also be determined by marking nuclei with a fluorescently tagged nucleoplasmic protein or a nuclear dye. In such cases, nuclear ROI can be selected by intensity-based thresholding. ImageJ plugin ‘3D objects counter’ can be used to semi-automate the process. In such cases, it is important to ensure that the NE deformation is accompanied by nucleoplasmic deformation (Figure 9A).

## Expected outcomes

The nucleus of a wild type budding yeast is circular. Perturbing pathways that contribute to maintenance of nuclear shape can result in dysmorphic nuclei (Deolal and Mishra, 2021). In a microscopy-based approach, the differences can be inspected visually. Such changes can be scored manually and reported as the fraction of cell population that has different morphology as compared to the wild type cells or the untreated cells. Additionally, such differences can be quantified as measurable parameters (Table 1). The method described here allows us to get a quantitative estimation of NE shape descriptors such as perimeter, area and circularity for marked ROI (Figure 9A)*.* The quantification method is easy to follow and does not require either expensive licensed software or coding based computational skills.

After obtaining the values of shape descriptor, here circularity index of nucleus and intensity-based line profile for ER, the comparison between samples should be done. Export the data obtained from step 10 to Excel. An example of nuclear outline in a mutant that affects nuclear shape is shown in Figures 7G and 7H. Final data showing a comparison of circularity index between wild type and *ssf1Δ* is shown in Figure 9A. Raw data and calculation are provided in additional excel files. In the attached excel file, we show data from a single field of wild type and *ssf1Δ.* The line profile shown in Figure 9C is a plot of cells shown in Figure 8D. The measurement is indicative of the intensity distribution along the length of selected ROI.

## Quantification and statistical analysis

At least 30 cells from each of the three biological replicates (total 3 × 30=90 cells) should be used for final quantification and statistical analysis. Significance is determined by using Student’s t-test to compare the differences in circularity index of wild type and *ssf1Δ* nuclei, assuming that the nuclei are marked randomly without any bias and data is distributed normally*.*

## Limitations

### Imaging

A limitation for live-cell imaging is the requirement for a suitable adapter and a temperature-controlled stage. If one does not have access to the live-cell imaging system, use manual focus or time point imaging by collecting samples at multiple points.

After 2–3 rounds of division, cells might begin to crowd. Also, non-adherent cells increase with longer imaging time (>8–10 h). This causes cells to float around and acquired images do not yield information. Such cells can be eliminated from analysis. If the organelle of interest has to be monitored for 2–3 rounds of division, select fields with 3–5, well-spaced cells in a field of 50 μm × 50 μm. If imaging time exceeds 8–10 h, immersion oil might dry, condensation of media on lids might be observed and nutrients can become limiting. Using sufficient media (1.5–2 mL) and starting with a dilute cell suspension would offset some of these issues. This is less of a problem when using glass dishes as compared to use of agarose pads. The agarose pads begin to dry after 2 h and exchange/ addition of media cannot be done.

Longer imaging (>4–6 h) can result in drift of field of view. However, minor drift can be corrected post-acquisition using microscope software or the ‘Manual drift correction’ plugin of FIJI/ImageJ.

### Image analysis

Although selection of ROI with free hand does not require images to be pre-processed and involves intuitive object picking, it has certain limitations. Due to manual intervention and heterogeneity of phenotype there can be bias in selection of ROI. However, this limitation can be overcome by imaging multiple fields across biological replicates and increasing the number of nuclei quantified.

For most of the abnormalities seen in the nucleus, calculating circularity index using an envelope marker is a good quantitative measure. However, the circularity index by itself cannot be used to bin/classify the abnormalities especially if the internal nuclear organization is also altered. In such cases, one can visualize the distribution of nucleoplasmic, chromatin and/or nucleolar markers (Figure 9A). First, the differences in the abnormalities can be inspected manually, followed by assessing measurable parameters. Further classification by assigning phenotypic categories, aggregation index, particle averaging and line profiles can also be used for quantifying differences between samples.

Semi-automated methods using correct edge detection methods yield more accurate selection of objects with continuous nuclear outline; but are difficult to obtain for discontinuous staining of the NE often seen in some mutants (Male et al., 2020-Figure 3A, row3). In such cases, multiple nuclear markers can be used. For large scale screens, semi-automated analysis or machine learning based training can be employed to get the better detection of objects with varying intensity and heterogeneity in phenotype**.**

## Troubleshooting

### Problem 1

Unclear field of view (Figure 3)

### Potential solution

Multiple uses of glass dishes can result in scratching of the cover glass base. This can affect the background. Use a new dish.

Dirt or suspended particles in either ConA or imaging medium can result in nonspecific signals. Prepare fresh stocks and filter sterilize before using.

If the field of view is crowded with cells or cells are layered, repeat step 6d until optimum density is obtained (Compare Figures 2B and 3A).

Ensure that there is sufficient immersion oil between objective and glass. Too little or excess immersion oil results in unclear focus.

### Problem 2

Lots of non-specific fluorescence/ high background (Figure 3)

### Potential solution

Cellular autofluorescence: We find that generally W303 gives more cellular autofluorescence than BY4741/42 due to adenine auxotrophy. Addition of additional adenine (1.5×–3×) to growth medium can help in reduction of autofluorescence or use *ADE2* version of W303. Avoid YPD if possible, as cells grown in YPD tend to give autofluorescence compared to cells grown in SC medium.

Non-specific fluorescent particles in the field of view: If there are any contaminating bacteria or dust particles in the imaging medium, you might see non-specific fluorescent signals (Figures 3B and 3C). Work in a sterile environment and use pure isolates of yeast strain.

### Problem 3

Not enough fluorescent signal

### Potential solution

Confirm the expression of protein by western blotting.

Marker protein can be expressed from a strong promoter in case of low protein expression from an endogenous promoter.

Make sure you are acquiring images with correct excitation/emission settings for the fluorophore of interest.

### Problem 4

Deconvolution introduces artifacts, or is not effective (Figure 6)

### Potential solution

Check Nyquist parameters (Figure 6A). Do not under/over sample.

Remove background using the Filtering or Unsharp mask of FIJI/ImageJ before deconvolving.

If the cells have high variation in the intensity of fluorescent signal, crop the image to a region of interest before deconvolving.

### Problem 5

The distance and intensity scale varies between samples

### Potential solution

Ensure that the pixel size of images used for analysis is same between samples

(To get image details, go to the Image menu and click on Properties).

Import images saved in original file format (along with metadata) to FIJI/ImageJ

(In order to use the Bioformats plugin, please refer to https://imagej.net/formats/bio-formats).

Compare the expression level of marker protein between samples by doing western blot.

## Resource availability

### Lead contact

Further information and requests for resources and reagents should be directed to and will be fulfilled by the Lead contact Dr Krishnaveni Mishra (krishnaveni@uohyd.ac.in).

### Materials availability

All reagents used have been cited in the key resources table. GFP-Esc1 and Pus1-GFP plasmid (Male et al., 2020) are available on request. Other reagents can be provided after obtaining permission from originally cited sources.

## Data Availability

This study did not generate new datasets. Original images if required are available on request.

## Supplemental information

Methods video S1. Coating the glass bottom dish and immobilizing the cells, related to step 3

## References

[bib12] Baker Brachmann C., Davies A., Cost G.J., Ca[puto E., Li J., Hieter P., Boeke J.D. (1998). Designer deletion strains derived fromSaccharomyces cerevisiae S288C: A useful set of strains and plasmids for PCR-mediated gene disruption and other applications.. Yeast.

[bib13] Belgareh N., Doye V. (1997). Dynamics of Nuclear Pore Distribution in Nucleoporin Mutant Yeast Cells.. J. Cell Biol. 13.

[bib14] Bevis B.J., Hammond A.T., Reinke C.A., Glick B.S. (2002). De novo formation of transitional ER sites and Golgi structures in Pichia pastoris.. Nat. Cell Biol..

[bib1] Deolal P., Jamir I., Mishra K. (2021). Uip4p modulates nuclear pore complex function in *Saccharomyces cerevisiae*. bioRxiv.

[bib2] Deolal P., Mishra K. (2021). Regulation of diverse nuclear shapes : pathways working independently, together. Communicative Integr. Biol..

[bib3] Janke C., Magiera M.M., Rathfelder N., Taxis C., Reber S., Maekawa H., Moreno-Borchart A., Doenges G., Schwob E., Schiebel E., Knop M. (2004). A versatile toolbox for PCR-based tagging of yeast genes: new fluorescent proteins, more markers and promoter substitution cassettes. Yeast.

[bib4] Longtine M.S., Mckenzie A., Demarini D.J., Shah N.G., Wach A., Brachat A., Philippsen P., Pringle J.R. (1998). Additional modules for versatile and economical PCR-based gene deletion and modification in Saccharomyces cerevisiae. Yeast.

[bib5] Lord C.L., Wente S.R. (2020). Nuclear envelope–vacuole contacts mitigate nuclear pore complex assembly stress. J. Cell Biol..

[bib6] Male G., Deolal P., Manda N.K., Yagnik S., Mazumder A., Mishra K. (2020). Nucleolar size regulates nuclear envelope shape in *Saccharomyces cerevisiae*. J. Cell Sci..

[bib16] Rapaport D., Brunner M., Neupert W., Westermann B. (1998). Fzo1p Is a Mitochondrial Outer Membrane Protein Essential for the Biogenesis of Functional Mitochondria in *Saccharomyces cerevisiae*.. J. Biol. Chem..

[bib8] Roggenkamp E., Giersch R.M., Wedeman E., Eaton M., Turnquist M.N., Schrock L., Jirakittisonthon T., Schluter-Pascua S.E., Bayne G.H. (2017). CRISPR-UnLOCK: multipurpose Cas9-based strategies for conversion of yeast libraries and strains. Front. Microbiol..

[bib17] Schäfer A., Kerssen D., Veenhuis M., Kunau W.-H., Schliebs W. (2004). Functional similarity between the peroxisomal PTS2 receptor binding protein Pex18p and the N-terminal half of the PTS1 receptor Pex5p.. Mol. Cell. Biol..

[bib9] Schindelin J., Arganda-Carrera I., Frise E., Verena K., Mark L., Tobias P., Stephan P., Curtis R., Stephan S., Benjamin S. (2009). Fiji - an Open platform for biological image analysis. Nat. Methods.

[bib18] Schindelin J., Arganda-Carrerass I., Frise E., Kaynig V., Longair M., Pietzsch T., Preibisch S., Rueden C., Saalfeld S., Schmid B. (2012). Fiji: an open-source platform for biological-image analysis.. Nat. Methods..

[bib10] Schuck S., Prinz W.A., Thorn K.S., Voss C., Walter P. (2009). Membrane expansion alleviates endoplasmic reticulum stress independently of the unfolded protein response. J. Cell Biol..

[bib19] Stade K., Ford C.S., Guthrie C., Weis K. (1997). Exportin 1 (Crm1p) Is an Essential Nuclear Export Factor.. Cell.

[bib20] Ulbrich C., Diepholz M., Baßler J., Kressler D., Pertschy B., Galani K., Böttcher B., Hurt E. (2009). Mechanochemical Removal of Ribosome Biogenesis Factors from Nascent 60S Ribosomal Subunits.. Cell.

[bib11] Wang Q., Xue H., Li. S., Chen Y., Tian X., Xu X., Xiao W., Fu Y.V. (2017). A method for labeling proteins with tags at the native genomic loci in budding yeast. PLoS ONE.

